# Deter or dispose? A critique of the relocation of asylum applicants to Rwanda and its public health implications

**DOI:** 10.1016/j.lanepe.2022.100442

**Published:** 2022-06-22

**Authors:** Jonathan Chaloner, Rebecca F. Baggaley, Bernard Ryan, Laura B. Nellums, Manish Pareek

**Affiliations:** a Lifespan and Population Health, School of Medicine, University of Nottingham, Nottingham, UK; b Department of Health Sciences, University of Leicester, Leicester, UK; c School of Law, University of Leicester, Leicester, UK; d Department of Infection and HIV Medicine, University Hospitals of Leicester NHS Trust, Leicester, UK; e Department of Respiratory Sciences, University of Leicester, Leicester, UK

For two decades the UK Home Office has instituted immigration policies to deter asylum seekers from entering the UK through irregular routes. The most recent is a Memorandum of Understanding (MoU) signed on 13 April 2022 to relocate asylum applicants in the UK to Rwanda, which would then be responsible for their asylum applications. This intention to discourage asylum seekers from crossing the Channel to seek safety implies the Home Office belief that asylum in Rwanda is undesirable.^1^ Though the European Court of Human Rights intervened to halt the first flight removing asylum applicants to Rwanda, the government remains committed to pursuing this policy, with plans underway for the next flight.

This policy perpetuates persistent rhetoric that the UK is being flooded by asylum seekers entering through ‘illegal’ routes. However, per head of population, the UK ranks 18^th^ in asylum applicants across the EU, EEA, and UK combined.^2^ Whilst asylum seekers in institutional contingency asylum accommodation in the UK have increased, this is due to the COVID-19 suspension of evictions, and long-standing delays in processing asylum applications, with over 50,000 individuals waiting six months or more for an initial decision.^3^ This has cost £1.5 billion per year, and £4.7 million per day on accommodation.^4^ Though the Prime Minister calls these costs to the taxpayer ‘unfair’, it can be argued they are driven by ‘fundamental failures of leadership and planning’ in the UK,^5^ rather than overwhelming migration.

Even with these costs, the financial justification for the MoU has been questioned. In an open letter to Home Secretary Priti Patel, Permanent Secretary Matthew Rycroft CBE raised his concerns about the policy's financial costs and value for money, which are dependent on it effectively deterring people smuggling, underscoring there was ‘not sufficient evidence’ that it would.^4^ Furthermore, the upfront costs of the policy are estimated to be approximately £30,000 per person; as Conservative MP Andrew Mitchell argues it ‘would be cheaper to put up those arriving in Britain at the Ritz for a year’ instead.

The MoU also raises significant human rights, public health, and legal concerns. The Home Office's ‘country policy and information note’ on the asylum system in Rwanda acknowledges Rwanda's lack of capacity to receive more asylum seekers or assess claims in a fair or timely way, with evidence of significant delays in decisions, discrimination, barriers to employment, and risks of exploitation, with particular concerns for asylum applicants with protected characteristics.^6^ The MoU also has cross-sectoral implications (e.g. for the Department of Health and Social Care (DHSC) and Rwandan Ministry of Health), including how the cultural and demographic differences of the relocated population compared to existing refugee populations in Rwanda (Figure 1) will be sensitively and appropriately accommodated, how the potential health risks to these populations in Rwanda compared to the UK will be mitigated (Figure 2), and the provision of health assessments and treatment, including who will be responsible for organising and paying for this, what long-term entitlement to care relocated asylum applicants will have, and to what extent care will align with global agreements and guidelines.

**Figure 1:**
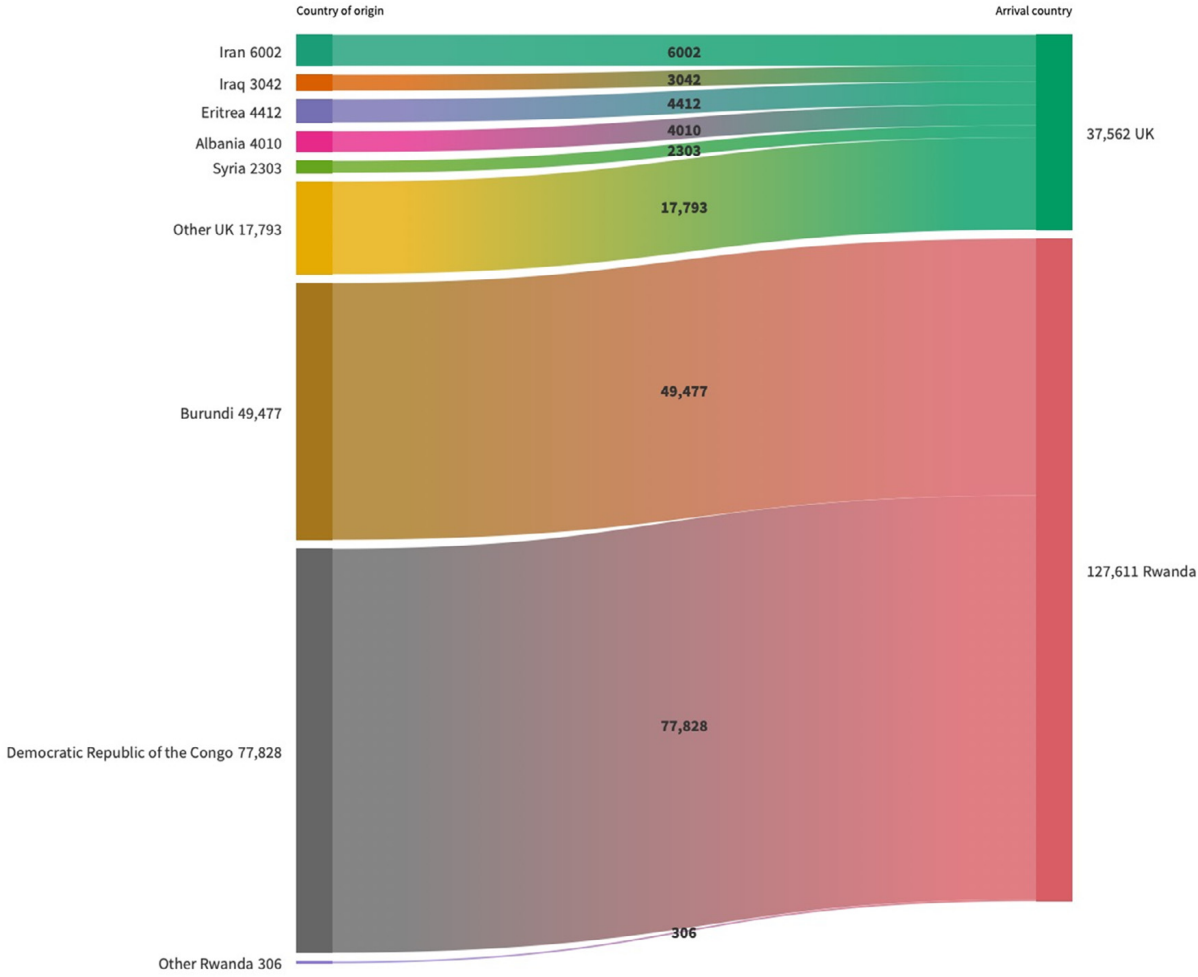
Asylum and refugee claims by country of origin for the UK and Rwanda in 2021.^8^^,^^9^

**Figure 2:**
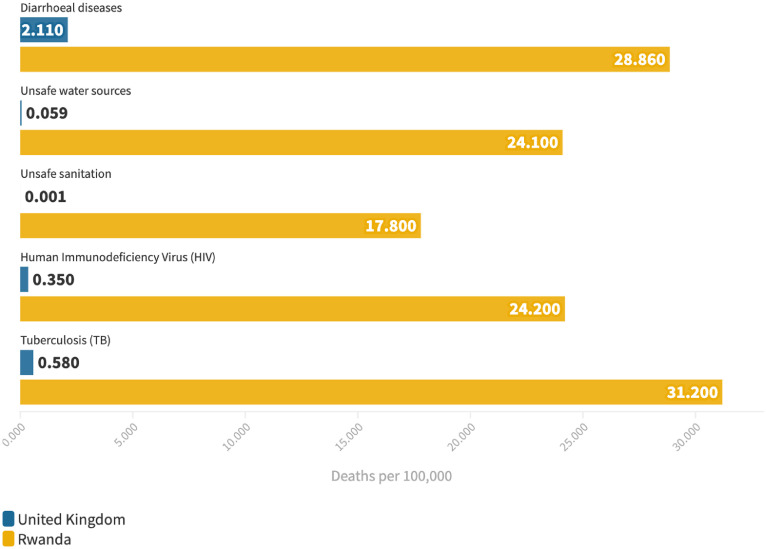
Mortality rates for key diseases of concern in asylum seeking and refugee populations in the UK and Rwanda.^10^

The timing of the MoU with COVID-19 also raises concerns, for example around the equitable delivery of vaccinations and risk of transmission in the asylum hostels to which migrants would be relocated, particularly in the context of Home Office failures to respond to numerous reports that their own accommodations were unsafe for migrants, leading to documented outbreaks.^5^ These populations are also at increased risk of depression, anxiety, and Post Traumatic Stress Disorder (PTSD), yet Rwanda has 0.06 psychiatrists per 100,000 compared to 6.4 in the UK.^7^

These public health risks link to legal concerns that sending asylum applicants to Rwanda would be incompatible with the UK's obligations to ensure effective international protection under the UN Refugee Convention, and to avoid inhuman and degrading treatment under the European Convention on Human Rights, alongside contraventions of British data protection law.

The MoU reflects an extreme version of a policy of deterrence and punishment of asylum applicants who arrive in the UK by irregular routes. This agreement will send these individuals to a so-called safe third country to which they are unlikely to have any previous connection, which will become responsible for their asylum applications. No other European state does this, not least because an EU directive precludes it. In the context of Brexit, however, this no longer applies to the UK, nor does the Dublin Regulation (which means France no longer has any obligation to take applicants back). States – including the UK – must have a humane policy on asylum seekers. The justification for this MoU must be interrogated urgently – both because of its legal, ethical, and public health implications, as well as the damaging precedent it will set for other countries that asylum is merely transactional and asylum applicants are disposable.

## Contributors

L.B.N., M.P., and J.C. conceived of the article. J.C. and L.B.N. wrote the first draft. All authors (J.C., R.F.B., B.R., L.B.N., and M.P.) contributed to subsequent drafts and the final version of the manuscript.

## Declaration of interests

MP declares grants to his university from Gilead, Sanofi, and Qiagen, and a consultancy with Qiagen, a molecular diagnostics company, to advise on a presentation about occupational health screening for latent tuberculosis. BR is an academic member of the Immigration Law Practitioner's Association. The other authors declare no competing interests.

## References

[1] Home Office (2022). https://www.gov.uk/government/publications/memorandum-of-understanding-mou-between-the-uk-and-rwanda/memorandum-of-understanding-between-the-government-of-the-united-kingdom-of-great-britain-and-northern-ireland-and-the-government-of-the-republic-of-r#part-4-financial-arrangements.

[2] UNHCR (2022). https://www.unhcr.org/uk/asylum-in-the-uk.html.

[3] Andy H. (2021). https://www.refugeecouncil.org.uk/wp-content/uploads/2021/07/Living-in-Limbo-A-decade-of-delays-in-the-UK-Asylum-system-July-2021.pdf.

[4] Rycroft M (2022). https://www.gov.uk/government/publications/migration-and-economic-development-partnership-ministerial-direction/letter-from-matthew-rycroft-to-rt-hon-priti-patel-accessible.

[5] Neal D. (2021). https://assets.publishing.service.gov.uk/government/uploads/system/uploads/attachment_data/file/1005065/An_inspection_of_contingency_asylum_accommodation_HMIP_report_on_Penally_Camp_and_Napier_Barracks.pdf.

[6] Home Office (2022). https://www.gov.uk/government/publications/rwanda-country-policy-and-information-notes/country-policy-and-information-note-rwanda-asylum-system-may-2022-accessible.

[7] World Health Organization (2022). https://www.who.int/data/gho/data/indicators/indicator-details/GHO/psychiatrists-working-in-mental-health-sector-(per-100-000.

[8] Home Office (2021). https://www.gov.uk/government/statistical-data-sets/immigration-statistics-data-tables-year-ending-september-2021#asylum-and-resettlement.

[9] UNHCR (2022). https://data2.unhcr.org/en/documents/details/90369.

[10] Institute for Health Metrics and Evaluation, University of Washington (2019). https://vizhub.healthdata.org/gbd-compare/.

